# A multi-depth spiral milli fluidic device for whole mount zebrafish antibody staining

**DOI:** 10.1007/s10544-023-00670-2

**Published:** 2023-08-15

**Authors:** Songtao Ye, Wei-Chun Chin, Chih-Wen Ni

**Affiliations:** 1 Quantitative and Systems Biology, University of California Merced, Merced, US; 2 Department of Bioengineering, University of California Merced, Merced, US

**Keywords:** Zebrafish embryo, Milli fluidic, Whole mount antibody staining, 3D printing

## Abstract

Whole mount zebrafish antibody staining (ABS) is a common staining technique used to localize protein information in a zebrafish embryo or larva. Like most biological assays, the whole mount zebrafish ABS is still largely conducted manually through labor intensive and time-consuming steps which affect both consistency and throughput of the assay. In this work, we develop a milli fluidic device that can automatically trap and immobilize the fixed chorion-less zebrafish embryos for the whole mount ABS. With just a single loading step, the zebrafish embryos can be trapped by the milli fluidic device through a chaotic hydrodynamic trapping process. Moreover, a consistent body orientation (i.e., head point inward) for the trapped zebrafish embryos can be achieved without additional orientation adjustment device. Furthermore, we employed a consumer-grade SLA 3D printer assisted method for device prototyping which is ideal for labs with limited budgets. Notably, the milli fluidic device has enabled the optimization and successful implementation of whole mount zebrafish Caspase-3 ABS. We demonstrated our device can accelerate the overall procedure by reducing at least 50% of washing time in the standard well-plate-based manual procedure. Also, the consistency is improved, and manual steps are reduced using the milli fluidic device. This work fills the gap in the milli fluidic application for whole mount zebrafish immunohistochemistry. We hope the device can be accepted by the zebrafish community and be used for other types of whole mount zebrafish ABS procedures or expanded to more complicated *in situ* hybridization (ISH) procedure.

**Graphical Abstract:**

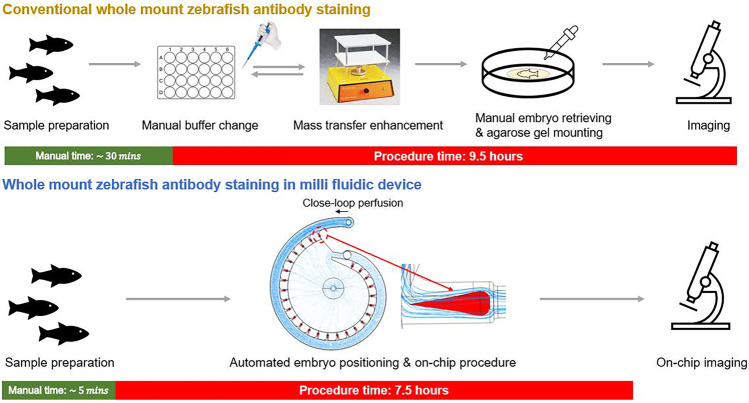

**Supplementary Information:**

The online version contains supplementary material available at 10.1007/s10544-023-00670-2.

## Introduction

Since the 1960s, zebrafish have served as one of the powerful and convenient small animal models in biomedical research. With its high reproduction rate, small size, body transparency, and closer phylogenetic relationship to humans, zebrafish coupled with molecular tools is ideal for high throughput *in situ* studies such as drug screening, embryotoxicity tests, genetic or tissue functional studies. However, most of the zebrafish related studies are still conducted manually in microplates or microtubes. Very little changes have been made in the last decades in the way of conducting zebrafish studies.

The whole mount zebrafish ABS and ISH using protein and antisense RNA probes are common molecular staining techniques for detecting the protein and gene localization information on zebrafish. The conventional manual procedure for the whole mount zebrafish molecular staining usually takes hours or days to complete and involves a series of tedious and time-consuming steps (Sorrells et al. 2013; Thisse and Thisse 2014). Moreover, the performance and consistency of the procedure is usually skill dependent. To overcome these limitations, automated liquid handling platforms have been developed to perform the assays. These platforms utilize robotic arms or hydraulic systems for automated liquid handling and are usually compatible with well-plate or tubes (Fuqua et al. 2021). Although they can greatly reduce the labor and improve the consistency of the assays, the platforms are not usually affordable in labs with limited budgets. In 2021, Fuqua et al. developed a semi-automated liquid handling platform, *Flyspresso*, for the whole mount fruit fly ABS. The *Flyspresso* utilizes a programmed gas-powered hydraulic system for the liquid handling which has a portable size and is compatible with various staining procedures (Fuqua et al. 2021). This open-source platform provides an economic way for developing customized automated platform for whole organism assays. However, it is not a “sample-in-and-answer-out” platform as the specimens still need to be retrieved and manually mounted for imaging. Moreover, the shaking or repeat pipetting-based mass transfer enhancements in tube and well-plate limit the room for process optimization and time reduction (Fuqua et al. 2021) To address these limitations, one way is to integrate the automated platform with fluidic devices.

In the last decades, several fluidic devices have been developed for ABS and ISH. Most of them are used for biomarker detection at cell and tissue levels (Brajkovic et al. 2018; Ciftlik et al. 2013; Huber et al. 2018; Kao et al. 2015; Maïno et al. 2019; Nguyen et al. 2017; Vanderhoeven et al. 2005). These devices have demonstrated that both sensitivity and specificity of the assay can be improved by the controlled microenvironments and the procedure can be speeded up by enhancing the convective mass transfer (Brajkovic et al. 2018; Ciftlik et al. 2013; Kao et al. 2015; Vanderhoeven et al. 2005). Despite these, the fluidic platforms for the whole organism ABS or ISH were rarely reported. In 2012, Akagi et al. developed a milli fluidic device that can automatically trap, immobilize and micro perfuse on lived zebrafish embryos. Integrating with a USB microscope, the system can be used for long term real-time *in situ* monitoring during fish embryo toxicity tests (FET) (Akagi et al. 2012). Notably, the whole mount zebrafish Trypan blue staining was performed using the milli fluidic device and staining process was found accelerated by applying high perfusion flowrate (Akagi et al. 2012). Although Trypan blue is not a macromolecular probe, the proof-of-concept study suggested that the mass transfer process can be enhanced with high flowrate even for the whole mount zebrafish. This study provides evidence that the whole mount zebrafish molecular staining procedure has the potential to be accelerated by using fluidic devices.

From engineering and economic perspectives, integrating the fluidic device, especially micro- or nano- fluidic with the automated platforms is indeed debatable. The main controversy lies in the complexity of the use, as well as the high cost associated with the device fabrication and development. Nevertheless, these concerns may not apply to milli fluidic devices. Compared with the conventional microfluidic device, the milli fluidic device that is used for whole organism studies usually has more resolution tolerance (Frey et al. 2022). Also, because the subject (i.e., whole organism) is at macroscopic level, applications such as trapping (Akagi et al. 2012), sorting (Panuška et al. 2021), drug dosage generation (Kao et al. 2015), etc. can be realized by applying relatively simple channel geometry and the results are less prone to defects (Frey et al. 2022). For these, the design and fabrication of the milli fluidic device can be achieved without the cleanroom. With the assistance of micro-milling machines (Akagi et al. 2012) or 3D printers (Fuad et al. 2017; Vanderhoeven et al. 2005), new channel designs can be rapidly prototyped and tested. Also, manufacturing processes such as injection molding and hot embossing can be easily adapted for mass production at low cost. Collectively, in contrast to the micro- or nano- fluidic device, the milli fluidic device will have more potential to be incorporated with the automated liquid handling platform and more likely to achieve the procedure automation level of “sample-in-and-answer-out”.

In this study, we developed a simple multi-depth milli fluidic device that can trap and immobilize the zebrafish embryo. The device is fabricated by a consumer-grade LCD SLA 3D printer assisted rapid prototyping method. For the first time, the complete procedure for the whole mount zebrafish ABS was performed and optimized on a milli fluidic device.

## Materials and methods

### Design and 3D printer assisted fast prototyping

The 2D layout of the milli fluidic device was generated by using AutoCAD (Autodesk Inc. San Rafael, California, USA) and then converted into 3D models in AutoCAD Fusion 360. The negative master model of the milli fluidic device was exported as an STL file and sliced in an Anycubic Photon Workshop slicer (Anycubic Inc. Shenzhen, China) for 3D printing. The master mold was printed by the Anycubic Photon Mono X LCD SLA 3D printer using the Anycubic gray UV resin. Briefly, the layer thickness was set to 50 µm with 16 seconds off time and 2 seconds UV exposure time to avoid the overcuring (Mohamed et al. 2019). After the 3D printing, the master mold was washed in 90% isopropanol for 30 mins and dried in the air. To prevent PDMS (Sylgard 184; DowCorning Corp, Midland, MI, USA) curing inhibition, the 3D printed master model was then UV exposed for 2 hours following a 16-hour heat treatment at 80 ^0^C (Venzac et al. 2021). For the PDMS soft lithography, the PDMS elastomer and curing agent were mixed at 10:1 (w/w) ratio and degassed before and after the PDMS casting to remove the air bubbles. The PDMS was then cured for 1 hour at 80 ^0^C and peeled off from the resin mold. For the tubing and accessory interconnection, 3 mm holes were punched in the PDMS layer with a biopsy punch (Robbins Instruments, Sunnyvale, California, USA). The PDMS layer was bonded with a manually cut microscope glass slide using an air plasma cleaner (Harrick Plasma Inc. New York, USA).

### Computational simulations

COMSOL 5.5 (COMSOL 5.5 Inc. Stockholm, Sweden) was used to perform the fluid dynamic and mass transfer simulations to evaluate the on-chip trapping and mass transfer processes. The “free and porous media flow” was used to simulate the steady state fluidic fields for the initial (i.e., no trap is occupied) and final (i.e., all traps are occupied) states of the trapping. The steady state fluidic field for the final state of trapping is then coupled with “the transport of diluted species in porous media '' to simulate the buffer replacement during the flushing process in a time-dependent study. The inlet flowrate was set to be 10 ml/min for both trapping and flushing simulations. Water was used as the carrying fluid in the simulations and the diffusion coefficient for the IgG antibody was defined as 1 $\times$ 10^–7^ cm^2^/s (Kao et al. 2015). To determine the maximum shear stress applied on embryos after trapping, the inlet flowrate was set to 20 ml/min as it is the maximum flowrate for the current system. The dechorionated zebrafish was molded as a cone-shape rigid body with 2 mm overall length and 0.5 mm maximum head diameter.

### System setup and operation procedure

A peristaltic pump (Kamoer Fluid Tech Co., Ltd, Shanghai, China) using a 2.3 mm ID and 4.6 mm OD polyurethane tube was used to drive the fluid in the system. To reduce the oscillation and smoothen the flow, a homemade pulse damper assembled by a 60 ml syringe and a two way-valve (Cole-Parmer Inc.) connected the chip outlet and the peristaltic pump inlet. A three way-valve was used to connect the peristaltic pump outlet, a 10 ml loading reservoir, and a waste container to allow both the close-loop perfusion and open-loop flushing. Silicone tubes with 2 mm ID and a 3 mm OD were used for the connection. This tubing size allows multiple embryos to be loaded and travel in the system simultaneously. Before operation, the fluidic device was first flushed with 70% (v/v) ethanol to wet the channel wall and prevent the bubble formation. The PBST buffer was used as the carrying buffer for the embryo trapping. To speed up the trapping process, the chip can be titled to let the gravitational force assist the trapping. For the consistency of the experiments, the flowrates for the trapping were set to be 10 ml/min. An acrylic M3 screw was used as the flow restrictor (FR) to partially block the main channel during the whole mount zebrafish ABS procedure (i.e., ON mode). Also, the FR was lifted during the trapping and flushing (i.e., OFF mode). When switching the buffers, the old buffer was first drained out into the waste container. The new buffer was then introduced into the system and flushing the channel in the open-loop for 30 seconds before switching back to close-loop perfusion. The flowrate used during buffer switching is 10 ml/min.

### Zebrafish husbandry, embryo UV treatment and fixation

**Fig. 1:**
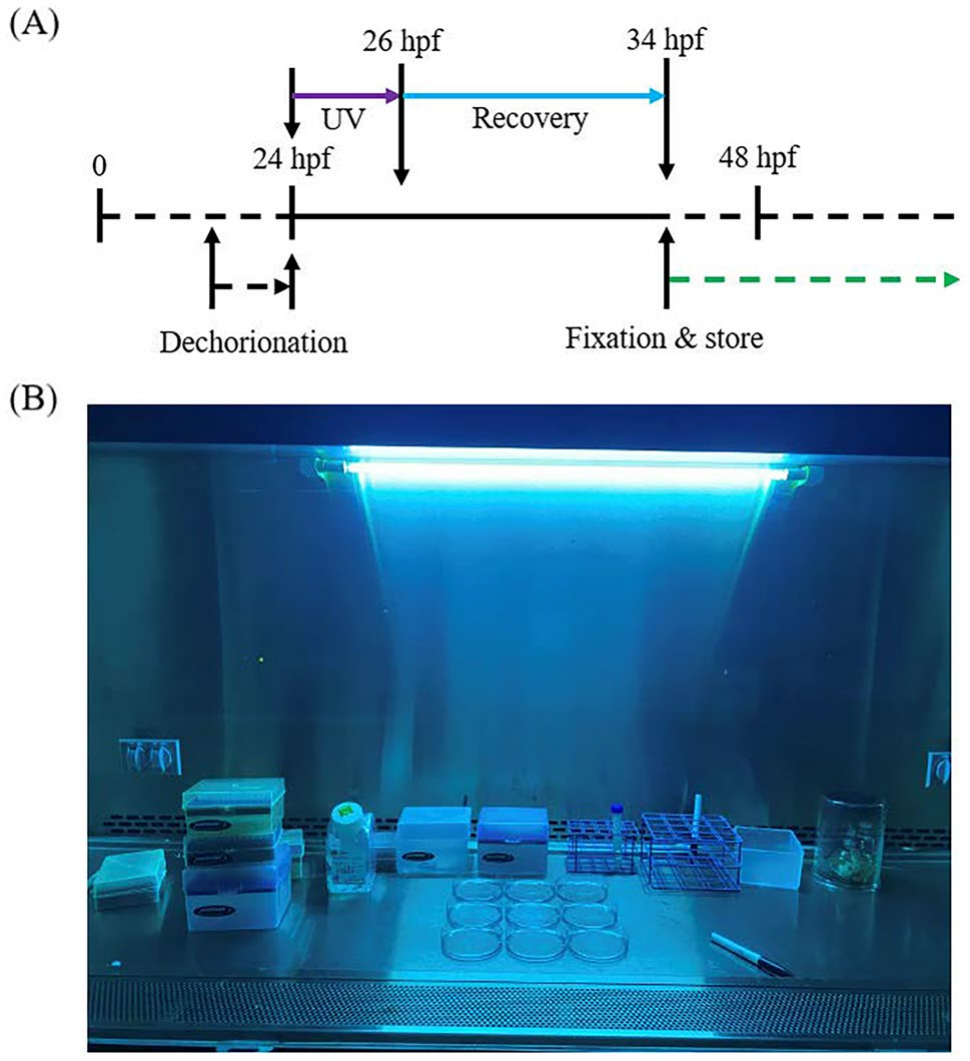
Zebrafish embryo Caspase-3 cleavage activation and sample preparation. **A** The schedule for the zebrafish embryo Caspase-3 cleavage activation treatments and sample preparation. **B** The image showing the dechorionated zebrafish embryos were under UV treatment inside the biosafety cabinet

Adult wild type zebrafish (EKW line) were raised in the UC Merced fish facility with a 14/10-hour light and dark switching cycle. The zebrafish were fed twice daily with dry feed (300 to 400 pellet size) to ensure healthy development. The zebrafish were randomly paired a night before the mating and spawning. The embryos were collected using a sieve and then rinsed with the E3 buffer to filter out the debris and wastes. The collected embryos were cultivated in the petri dish filled with E3 buffer at 28.5 ^0^C and dead embryos were sorted during the development. To activate the Caspase-3, 24 hpf embryos were manually dechorionated and then transferred into a petri dish (~ 100 embryos per dish) filled with 15 ml E3 buffer. There are two reasons why we dechorionate the zebrafish embryo. First, the zebrafish chorion contains Gadusol a natural sunscreen that can protect the embryo from UV induced damage (Rice et al. 2023). Therefore, dechorionation is necessary to maximize the effect of later UV treatment. Second, the dechorionated embryo makes the later imaging easier as different part of the embryo can be targeted for signal measurements. After the dechorionation, the chorion-less embryos were then exposed under the UV lights (254 nm, ~ 100 µW/cm^2^) in the biosafety cabinet (NuAire Inc. Plymouth, USA) for 2 hours following 8 hours recovery in an incubator at 28.5 °C (Zang et al. 2022). The treated embryos were fixed in 4% formaldehydes/PBST for overnight at 4 °C and then stored in 100% methanol at – 20 °C for later staining, Fig. 1A, B.

### Whole mount zebrafish Caspase-3 antibody staining

The procedures for the plate-based and device-based whole mount zebrafish Caspase-3 ABS are listed in Table 1. The sample preprocessing for both plate-based and device-based procedures are performed in the 24-well plate. A belly-dancer (IBI Scientific Inc., Iowa, USA) was used to provide medium level shaking for the on-plate steps. For the plate-based whole mount zebrafish Caspase-3 ABS, 10 to 15 embryos were loaded per well in the 24-well plate. For the device-based whole mount zebrafish Caspase-3 ABS, the number of embryos tested on the device was dependent on the trapping result. The flowrate and time for each on-chip staining step was kept constant at 10 ml/min and 2 hours, respectively. The dilution ratios for the primary antibody and secondary antibody were kept at 1:1000 (Cell signaling technology #9661, -Caspase-3, Rabbit) and 1:200 (Invitrogen #31,686, -Rabbit Rhodamine, 0.5 mg/ml in stock), respectively.

**Table 1:**
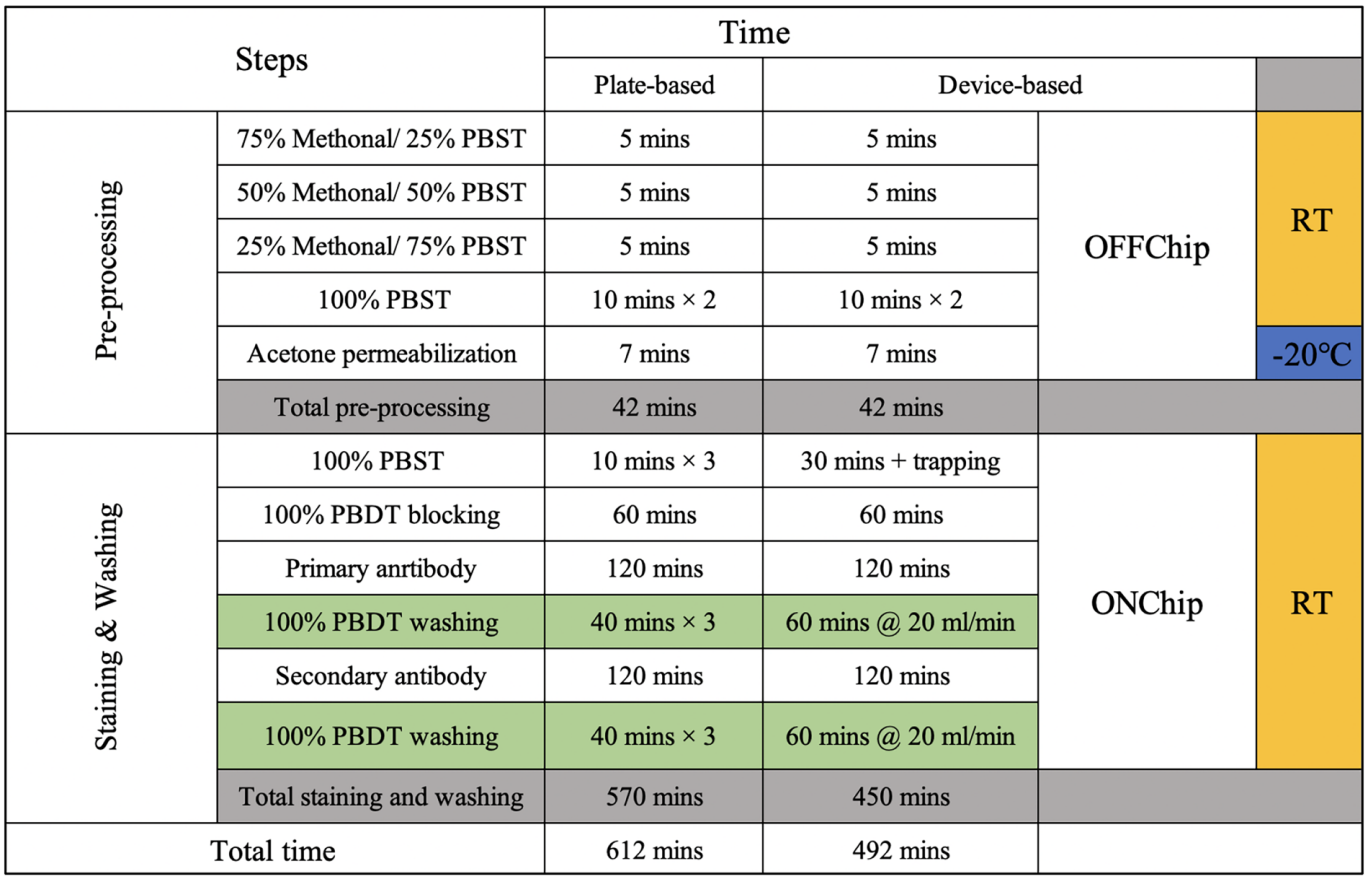
Whole mount zebrafish Caspase-3 ABS procedures

### Imaging and signal quantification

The fluorescence images were acquired under the confocal microscope (Leica Biosystem Inc.) with 100 $\times$ magnification, 15% laser power, and 800 V digital gain. The laser beam scanned the zebrafish embryo along the Z direction every 5 µm to get the sliced images. The sliced images were then stacked to form a Z projected image using the maximum intensity. ImageJ was used to analyze the signal levels on the Z-projected images by applying masks with threshold level of 100 on the 8-bit gray scaled images.

### Data analysis and reproducibility

The statistical analysis was conducted by GraphPad Prism (GraphPad Software) and MS Excel (Microsoft, USA). The Student T-tests were used to measure the significant level (P < 0.5) between the groups. To ensure unbiased experimental results, the controlled experiments were conducted for at least 3 replicates (i.e., N $\ge$ 3) with at least 10 embryos per replicate (i.e., n $\ge$ 10). The results were measured and quantified under the same standard. Embryos collected from the same batch were only to be used for the same type of experiments. To avoid the risk of contamination, the PDMS-glass devices were only for one time use and all the buffers were used within the recommended storage periods.

## Experimental results

### Device design and fabrication

**Fig. 2:**
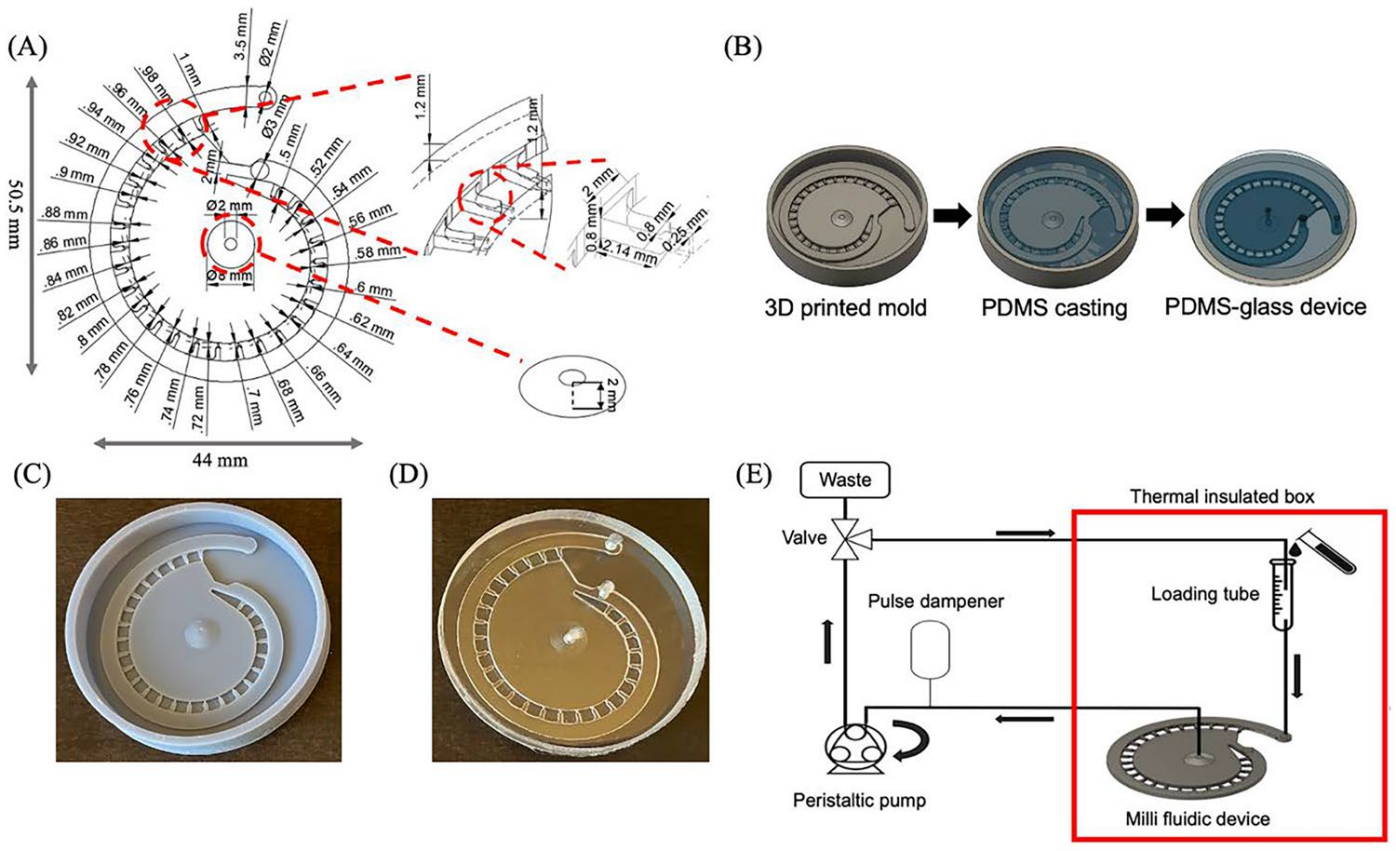
Multi-depth spiral milli fluidic device for zebrafish immobilization and antibody staining. **A** The engineering drawing showing the dimension of the multi-depth spiral device. **B** The 3D printing assisted prototyping process. **C** Photography showing the 3D printed negative master mold for the multi-depth spiral device. **D** Photography showing the assembled PDMS-glass device after soft lithography. **E** Schematic showing the system setups. The arrows indicate the flow directions

A multi-depth spiral device was designed to trap, immobilize, and perform the whole mount ABS on the 24 to 48 hpf chorion-less zebrafish embryos. The device contains 3 major functional parts: a spiral main channel, an inner suction chamber, and 26 trapping channels that interconnect the main channel and inner reservoir, Fig. 2A. Hydrodynamic suction was used as the main trapping and immobilization force in this design. When operating, the inner chamber, which connects the peristaltic pump at the outlet, provides a negative pressure to draw the fluids from the main channel via the traps. The height for the main and the trap channel is 1.2 mm and 0.8 mm, respectively. The funnel-like outlet has a peak height of 2 mm, a 2 mm ID and an 8 mm OD. The purposes for applying multi-depth design in the trap, main channel, and outlet are: 1) to shorten the mass transfer distance in the traps; 2) to prevent bubbles from entering the traps; 3) to release the bubbles accumulating in the inner reservoir. Also, the main channel has a width of 3.5 mm which allows multiple chorion-less embryos to travel simultaneously and let individual embryo to freely self-orientate. The trap entrance has a width of 2 mm, a length of 2.14 mm, and the width of the trap nozzle is 0.25 mm. The nozzle length is used to regulate the strength of the hydrodynamic suction force during trapping. The length of each individual trap is decreasing from 1 mm to 0.5 mm at a rate of 0.02 mm/trap along the main channel. This configuration is to ensure a close hydrodynamic trapping effect or embryo drawing ability at each trap for the purpose of maximizing the trap usage, smooth the trapping process, and minimize the procedure lagging, Fig. 2S. A 3 mm diameter flow restrictor (i.e., an acrylic M3 screw) is used to partially block the end of the main channel during the whole mount zebrafish ABS. The total internal volume of the device is about 1 ml which is close to the volume commonly used at a single well in a 24-well plate. Besides, the internal volume of individual trap is about 1.37 $\mu l$ which ensures “one embryo per one trap” and a high surface-to-volume environment for the embryo. To reduce the flow pulsing caused by the peristaltic pump, a homemade pulse dampener, customized by a 60 ml lure head syringe, is connected in between the device outlet and the peristaltic pump, Fig. 4S. The minimum operating volume is about 6 ml which allow the flow to continuously circulate in the system without any vacant space (i.e., air volume). Furthermore, the system can switch between close-loop and open-loop perfusion by adjusting the 2-way valve, Fig. 2E.  In addition, embryos can be loaded into the system all at once for close-loop trapping, thanks to a spacious main channel used in the milli fluidic chip design. This feature provides our system with an advantage in terms of embryo loading convenience over some previously reported zebrafish embryo  trapping systems, which could only load one embryo at a time (Akagi et al. 2012; Fuad et al. 2017).

The PDMS-glass device in this work was fabricated by using the LCD SLA 3D printer assisted fast prototyping method, Fig. 2B. The negative mold of the device was printed by the Anycubic Photon Mono X LCD SLA 3D printer, Fig. 2C. After printing, the mold was washed with 70% IPA and dried in the air. To prevent the PDMS curing inhibition, the 3D printed mold was post-treated with UV light for 2 hours following 16 hours heat treatment at 80 ^0^C (Venzac et al. 2021). The PDMS was then casted onto the 3D printed mold and cured at 80 ^0^C for 1 hour. After curing, the PDMS layer was peeled off from the mold and plasma bonded with a microscopic glass slide, Fig. 2D. The LCD SLA 3D printer used in this work uses a 3840 2400 (4 K) LCD screen and an array of UV lamp beads to control the pattern printing at each layer. To avoid overcuring and resin flow disturbing, the UV exposure time at each layer was set to 2 seconds with a 0.25 mm/s lifting speed and a 3 mm/s retract speed. The 3D printer employs an LCD screen with 50 µm by 50 µm pixel size that cannot always fit the edge of design perfectly. Therefore, jagged channel walls are expected to be casted using the mold printed by LCD SLA 3D printer. To reduce the edge jaggedness of the mold, the anti-aliasing algorithm was also applied in the settings. To evaluate the fabrication quality, the nozzle width is inspected as it is the smallest feature in the device. The result has indicated that the average nozzle width is about 0.2850 ± 0.031 mm (N = 5) when the target width is 0.25 mm, Fig. 1S. As expected, the channel wall was also found to be rough, even applied with the highest level of anti-aliasing. The variation of the channel width and the rough channel wall were considered acceptable as only a limited hydrodynamic difference could be caused by the small variations and the elastic property of the PDMS would extenuate the mechanical damage on the zebrafish embryo.

### Embryo trapping and immobilization

**Fig. 3:**
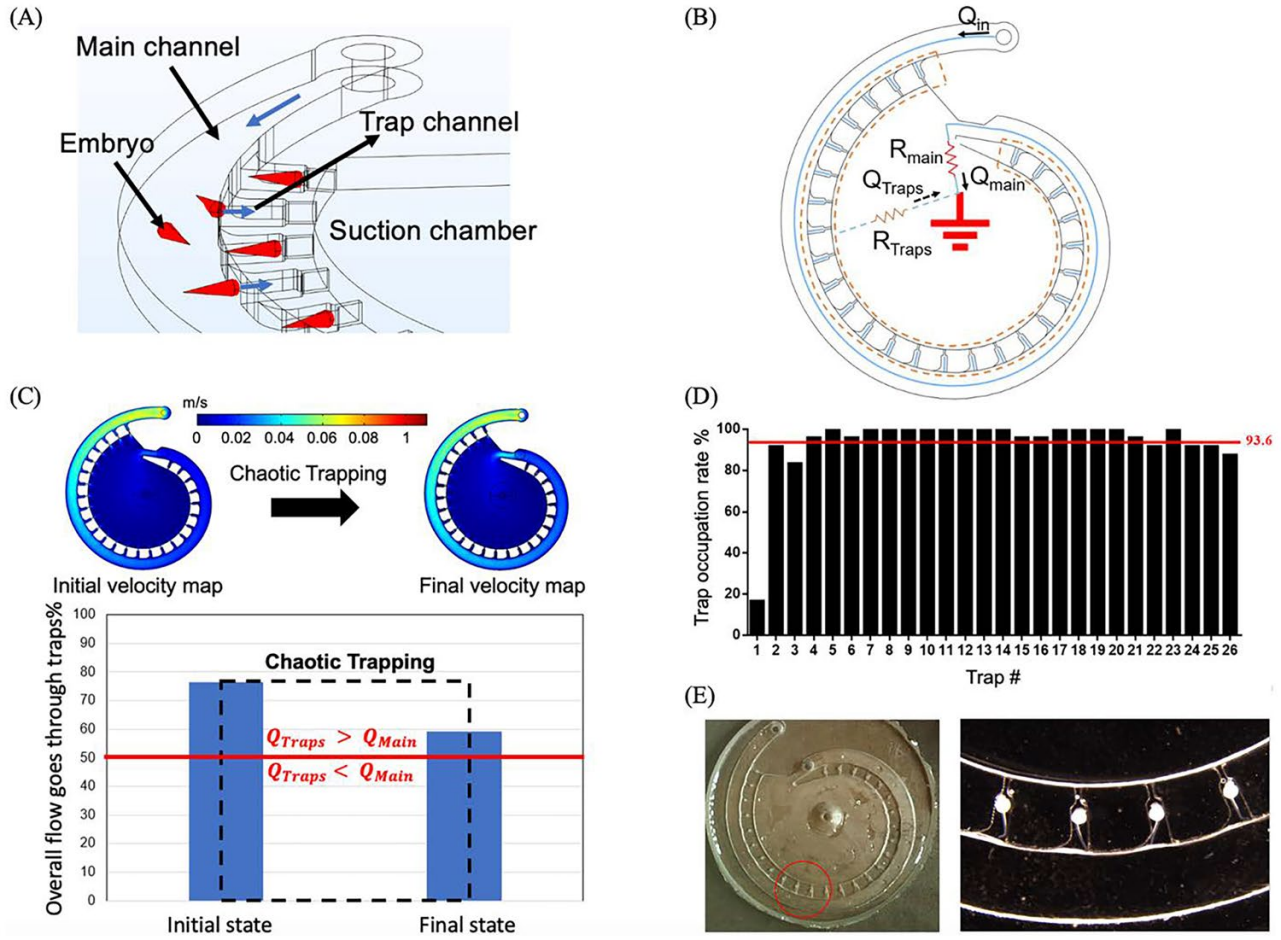
The zebrafish embryo trapping principles and validations. **A** The schematic showing the torpedo-shaped embryos are dragged into the traps due to the hydrodynamic suction force. **B** The simplified circular analogy for the multi-depth spiral device showing the parallel trapping and source and sink configurations for the design. **C** The CFD simulations for initial and final fluidic conditions for the trapping process. The possibility for the embryo trapping retains during the trapping process as the overall flowrate goes through the traps is always higher than the flowrate passes through the main channel. Inlet flowrate is set to be 10 ml/min. **D** The trap occupation rate for each individual traps (N= 24, n=624). The red line indicates the overall average trap occupation rate. 36 hpf UV treated embryo with body length around 2 mm are used in the trapping validation experiments. **E** Microscopy images showing the zebrafish embryos are encapsulated by the PBST droplets inside the traps due to the presence of the Laplace pressure

During trapping, the hydrodynamic suction force deviates the embryos towards the inner wall and then drags the embryos into the traps, Fig. 3A. The hydrodynamic trapping process can be easily explained by a simplified circular analogy diagram, Fig. 3B. Briefly, the embryo trapping occurs, when the overall flow that passes through the traps is greater than the flow that travels through the main channel (Q_Traps_ > Q_main_). In this chip design, the traps are arranged in a parallel configuration (Nguyen et al. 2016) and the inner reservoir can be treated as the ground that provides a relatively constant downstream pressure, Fig. 2S. Based on the CFD simulation, the overall flow that passes through the traps (i.e.,Q_Traps_/Q_in_ × 100%) drops from 76%, when all traps are empty (i.e., initial state), to about 59%, when all the traps are occupied by the 2 mm cone-shaped embryos (i.e., final state). The simulation provides an intuitive understanding of the embryo trapping process in which the trapping become increasingly difficult as more embryos are captured by the traps. Despite this, the possibility for the embryo trapping remains at the last moment of the trapping process as the flow passing through the traps is still higher than the flow going through the main channel, Fig. 3C.

In the trapping validation tests, 26 embryos were loaded into the device at each trial. For the consistency, the overall trapping process only allows for about 5 mins in the close-loop. To maximize the usage of the traps, gravitational force was introduced to assist the trapping at the late trapping phase (i.e., most traps are occupied) in which the device was titled towards the remaining empty traps (Movie S1). When applying 10 ml/min for close-loop trapping, the trap occupation rate can easily reach over 84% without gravitational force (Movie S2, data not shown) and can achieve 93.6 ± 4.0% (N = 24, n = 624) with the assistance of the gravitational force, Fig. 3D. The trapping results also indicate that the first trap was usually skipped by the embryos even with the help of the gravitational force. This is due to the high main channel velocity near the inlet which gives the embryo a less time to deviate towards the inner wall. Therefore, the first trap in this design mostly plays the role of the divergent channel to enhance the inner wall deviation of the embryos. The orientation control of the zebrafish embryo was not in the design considerations initially as a confocal microscope is used in the signal measurement. Interestingly, the validation experiments show that about 93.7 ± 4.3% (N = 24) trapped embryos had their heads pointing inward after the chaotic trapping. We believe this orientation consistency is contributed by the coupling effects of chorion-less embryo’s the body shear stress distribution and the hydrodynamic suction. Briefly, when traveling in the main channel, the body shear stress is nonuniformly distributed on the embryo which makes the embryo have the tendency to rotate. Meanwhile, the hydrodynamic suction draws the embryo towards the inner wall traps, and the embryo’s head is likely to point inward as the head experiences a stronger drag force than the narrowed body. Hence, in compared to the narrowed body, the head of embryo is more likely to be dragged by the trap, Fig. 3S.

The fully liquid-filled device makes transportation difficult as the flow disturbing (e.g., backflow) caused by actions such as unplugging tubes and shaking or sudden movements can displace the embryos from their positions. For this, the buffer is drained out of the device before transportation. Interestingly, due to the presence of Laplace pressure, the holdup buffer volume will form isolated droplets and wrap around individual embryos inside the traps, Fig. 3E. These isolated droplets will not only prevent the zebrafish embryo from displacing during transportation, but also provide liquid immersed environment for the encapsulated zebrafish embryos during imaging (i.e., keep sample hydration and increase the imaging resolution). This feature makes the device portable and allow it to be directly transported and used in various imaging platforms. In terms of stability, none of the embryos was displaced during the device transportation. To our best knowledge, this feature has not been reported by other similar devices. Other potential usages for this feature in zebrafish studies such as metabolism analysis, hypoxia studies, and toxicity tests may be worth investigating in the future.

### On-chip flow settings

**Fig. 4:**
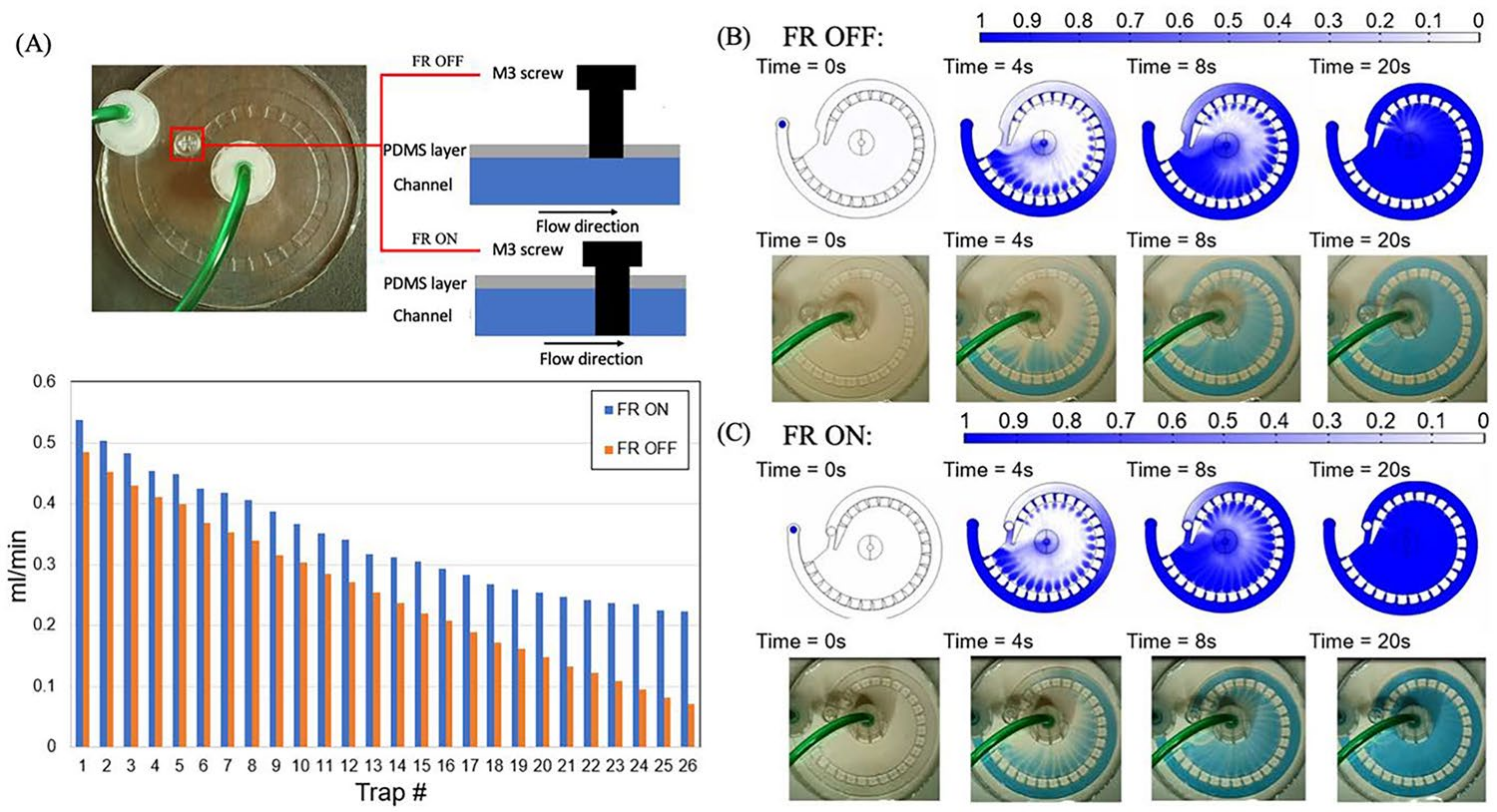
On-chip flow settings for the multi-depth spiral device. **A** Top: The flow restrictor (FR) ON and OFF modes for the device. Bottom: The flowrates at each individual traps at FR ON (blue) and FR OFF (orange) modes. **B** The perfusion simulation (top) and validation (bottom) for FR OFF mode at 10 ml/min inlet flowrate. **C** The perfusion simulation (top) and validation (bottom) for the FR ON mode at 10 ml/min inlet flowrate

The multi-depth spiral device uses a simple parallel trapping configuration. However, the limitation for this configuration is also obvious as the flowrate in the individual traps drops along the spiral main channel (Nguyen et al. 2016). This is not ideal for the on-chip ABS because the convective mass transfer rate is now different at each trap and the last trap always takes the longest time to complete the mass transfer. To minimize this procedure lagging, a flow restrictor (FR) is added to partially block the main channel during the on-chip ABS. Based on the CFD simulation, the add-on of the FR can boost the flowrate in the traps as well as close the flowrate differences among the traps, Fig. 4A. The validation experiments using the methylene blue/PBST also confirmed that the FR ON mode enhances the perfusion in the traps as it took less time for the dye to fill the traps, Fig. 4B, C. Here, the flowrate for both the CFD simulations and validation experiments was set to 10 ml/min.

**Fig. 5:**
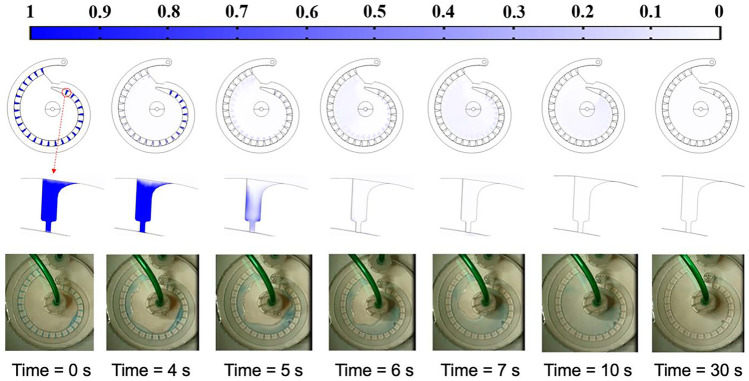
The flushing time estimation. **Top panel**: the overall mass transfer simulation for the flushing process. **Middle panel**: mass transfer simulation for the last trap (i.e., 26^th^ trap) during open-loop flushing. **Bottom panel**: the flushing validation experiment using methylene blue/PBST. The flushing flowrate for both simulation and experiment are 10 ml/min

During the buffer switching (Movie 3S), the old buffer will first be flushing out in the system. To ensure the overall system is replaced by the new buffer and all the holdup volume inside traps is cleaned up, the system is then flushed with the new buffer in the open-loop before switching to the close-loop circulation. In the simulation, the old buffer (i.e., the PBDT with antibody) was set to be replaced by the new buffer (i.e., PBDT) at 10 ml/min. The simulation showed that it takes about 7 seconds for new buffer to reach and flush out the old buffer in the last trap, Fig. 5, middle panel. However, the device is not completely cleaned as the old buffer is still accumulated at the center chamber, Fig. 5, top and bottom panels. To completely flush out the old buffer from the system, the flushing process needs to carry on for another 23 seconds to an overall 30 seconds flushing time. Both simulation and validation experiments using methylene blue/PBST have shown that the system can be completely replaced by the new buffer after flushing for 30 seconds at 10 ml/min (Movie 4S). Note, during the buffer switching, the FR is turned OFF (i.e., M3 is lifted) to prevent the potential bubble blocking.

**Fig. 6:**
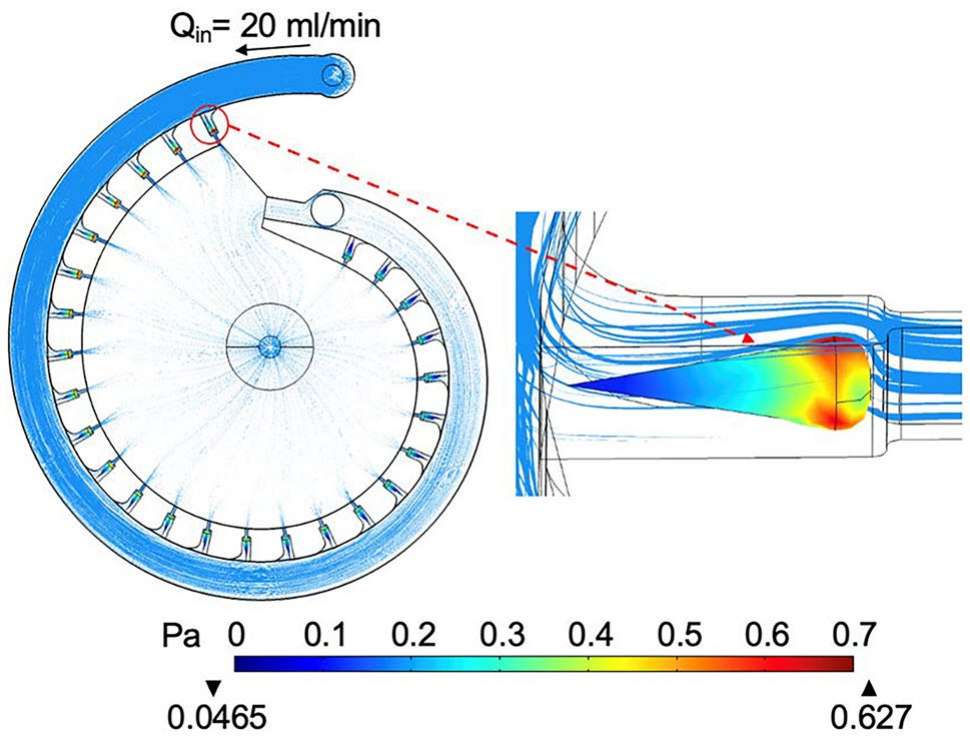
The simulated body shear stress on the fixed zebrafish embryos during on-chip perfusion. The embryo in the first trap is selected to show the maximum shear stress level and the shear stress distribution. The flowrate is set at 20 ml/min and the FR is turned on

One of the concerns for on-chip flowthrough operations is the body shear stress exerted by the superficial flow may damage the fixed embryos. The CFD simulation showed that when perfusion flow is at 20 ml/min and the FR is turned ON, the maximum shear stress applied on the embryo is about 0.672 Pa and the shear stress hot spot is located at the upper surface of the embryo, Fig. 6. We believe this shear stress level is unlikely to damage the fixed zebrafish embryos as tissue becomes more rigid and less fragile after formaldehyde fixation. Indeed, most observed embryo damages were during the off-chip pipetting and embryo transferring.

### Multi-depth channel for bubble prevention

The air bubble introduction is the major cause of biases for the device-based zebrafish assays. Bubbles can obstruct the trap during the perfusion, interfere with the embryo imaging, and induce backflow, all of which can adversely affect the accuracy and reliability of the assay results. As most PDMS-glass milli fluidic devices are integrated with open pumping systems, the air may enter the device through both inlet and the gas permeable PDMS layer leading to the formation of liquid–gas flow in the device. Likewise, in our current system setup, the flow is driven by a peristaltic pump. To reduce the pulsing caused by the peristaltic pump, the buffer loading tube must open to the air. Therefore, air can potentially be introduced into the device during the operation. By far, the only attempt to prevent the air bubbles in the milli fluidic device is made by Zhu et al. (2019). The group reported a bubble free milli fluidic device by bonding a PDMS vacuum layer on top of the PDMS zebrafish embryo culture channel layer (Zhu et al. 2019). In their study, the negative pressure in the vacuum chamber can effectively remove the bubbles from the embryo culture channel. However, the multilayer PDMS device will complicate the fabrication steps and the additional PDMS layer may affect the signal detection during the imaging. Moreover, the application of external negative pressure may affect the zebrafish embryo culture environment such as oxygen level. Here, we demonstrated a simple channel geometry-based bubble prevention method for the single layer milli fluidic device.

**Fig. 7:**
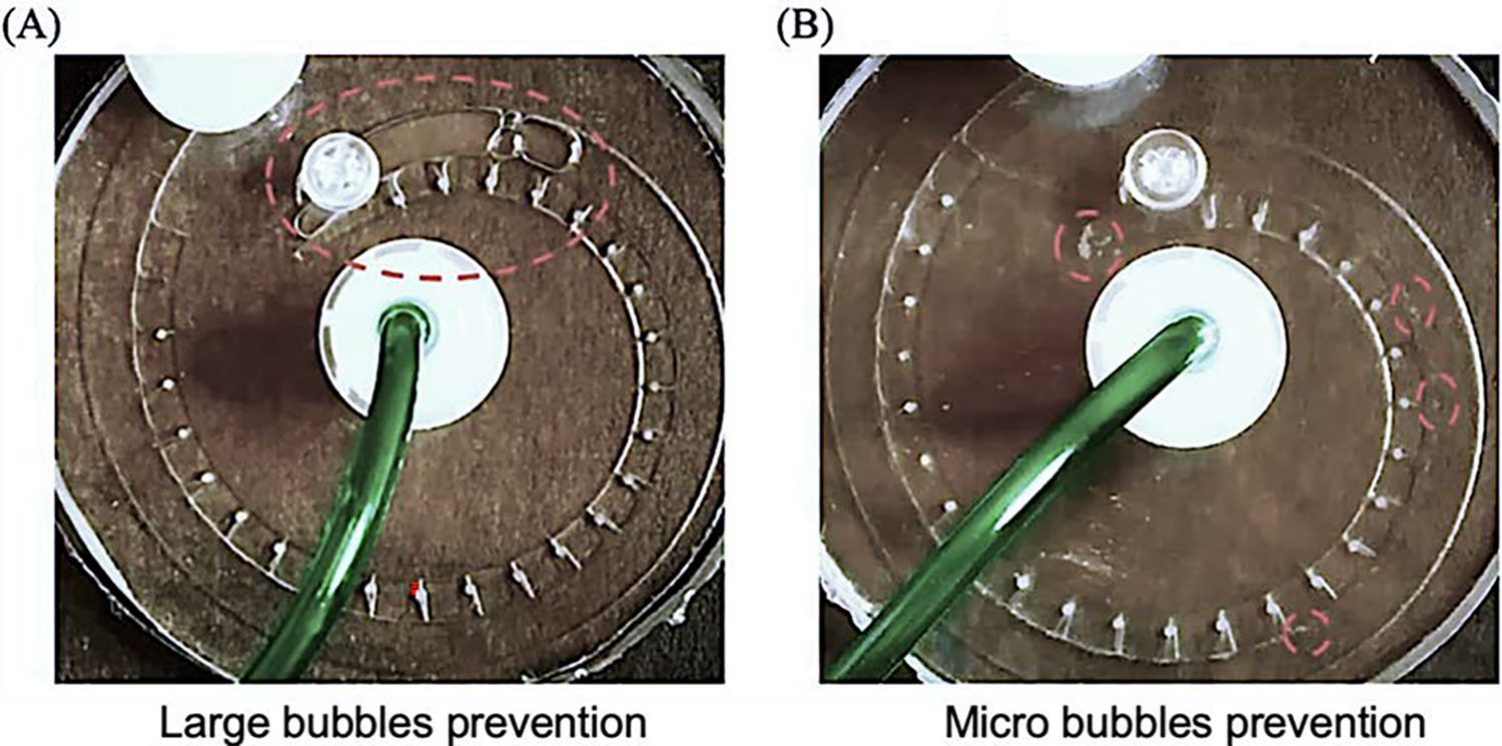
Bubble prevention in the milli fluidic device. **A** The large bubble formed during buffer switching can be easily flushed out by increasing the flowrate or slightly tilting the device. **B** The micro bubbles formed at high perfusion flowrates are traveling near the top surface of the main channel and not entering the traps due to the channel height difference

In our current system setting, buffer switching, and high flowrate perfusion are the two situations where most bubble introduction events occur. Nevertheless, the types of bubbles that are introduced in these two situations are different, as are the underlying bubble formation mechanisms. During the buffer switching step, the channel was first emptied and the new buffer together with air was then perfused into the channel. The bubbles introduced during the buffer switching step are usually large or medium sized bubbles, Fig. 7A. These bubbles which have size larger than the trap are unlikely to enter the traps during the perfusion. However, the movement of the large bubbles may cause pressure fluctuations, induce backflow, and as a result disturb the trapped embryos. Our milli fluidic device offers a spacious main channel for the large bubbles which can minimize the pressure fluctuations caused by their movements and thus unlikely to disturb the trapped embryo. Furthermore, because of their large radius, the large air bubbles experience less capillary retaining force in the main channel and therefore can be easily flushed out of the device (Movie 5S). Note the narrow section at the end of the main channel may prevent the large bubbles from being removed. Increase the flushing flowrate or slightly title the device can help to remove the large bubbles stuck at the end of the main channel.

When operating at the high perfusion flowrate (i.e., 20 ml/min), micro bubbles were found in the channels, Fig. 7B. This is because air was introduced with the buffer at high pumping speed generating dispersed-bubble flow in the channel. Besides, the use of detergents such as Triton X-100 and Tween 20 also aggravates the formation of the micro bubbles. In our device, because the main channel height is greater than the trapping channels, micro bubbles travel near the top surface of the main channel and are unlikely to enter the traps (Movie 6S).

### Optimization of the on-chip whole mount zebrafish Caspase-3 antibody staining

The whole mount zebrafish Caspase-3 ABS is a well-established assay to detect the level of cell apoptosis in the zebrafish (Sorrells et al. 2013). To induce the Caspase-3 cleavage, we applied UV light, a common environmental stress triggering apoptosis pathway, on the zebrafish embryo (Zeng et al. 2009). To investigate if the whole mount zebrafish ABS can be improved in the flowthrough environment, we next tested the whole mount zebrafish Caspase-3 ABS on the fluidic device and compared the performance with conventional plate-based manual procedure. The procedures for the plate-based and device-based whole mount zebrafish Caspase-3 ABS are shown in Table 1. Note the washing time and flowrate shown in device-based procedure is the final optimized results. The standard on-plate washing time is 120 mins.

The general whole mount zebrafish ABS procedure involves both staining and washing steps (Sorrells et al. 2013). To ensure the antibody can sufficiently bind to the antigen, the staining time is usually kept at an extended level (e.g., overnight). Despite no systematic study has been conducted to investigate how the fluidic flow can affect the antibody-antigen interaction in the whole mount zebrafish, the studies performed in tubes or well-plates suggested that the antibody staining took longer in older embryos as the tissue became denser (Sorrells et al. 2013). The intact tissue of the zebrafish embryo certainly affects the antibody penetration. Therefore, optimizing the whole organism staining procedure by reducing the staining time may result in loss of sensitivity. The washing step, on the other hand, is for the removal of the nonspecific binding after the staining step and is crucial for the specificity of the procedure. Targeting the washing steps will be a safer option for the optimization of the whole organism staining as the true signal is ensured with sufficient staining time. Also, the manual buffer refreshing steps can be avoided during on-chip washing as the wash buffer is circulated in a close-loop. For these, the washing steps were targeted for the on-chip whole mount zebrafish Caspase-3 ABS optimization. Briefly, in the milli fluidic device, two flowrates viz., 10 ml/min and 20 ml/min were used to perform 30 mins, 60 mins, 90 mins, and 120 mins washings after each staining step, Fig. 6A, top panel. To ensure the consistency and sufficient Casapse-3 binding, the flowrate and time for the two staining steps were kept constant at 10 ml/min and 120 mins, respectively. For comparison, the same washing times were tested in the plate-based procedure using a 24-well plate. After the procedure, the Caspase-3 signals were measured from the zebrafish embryos encapsulated by the PDST droplets in the milli fluidic device and 0.5% agarose in well-plate.

**Fig. 8:**
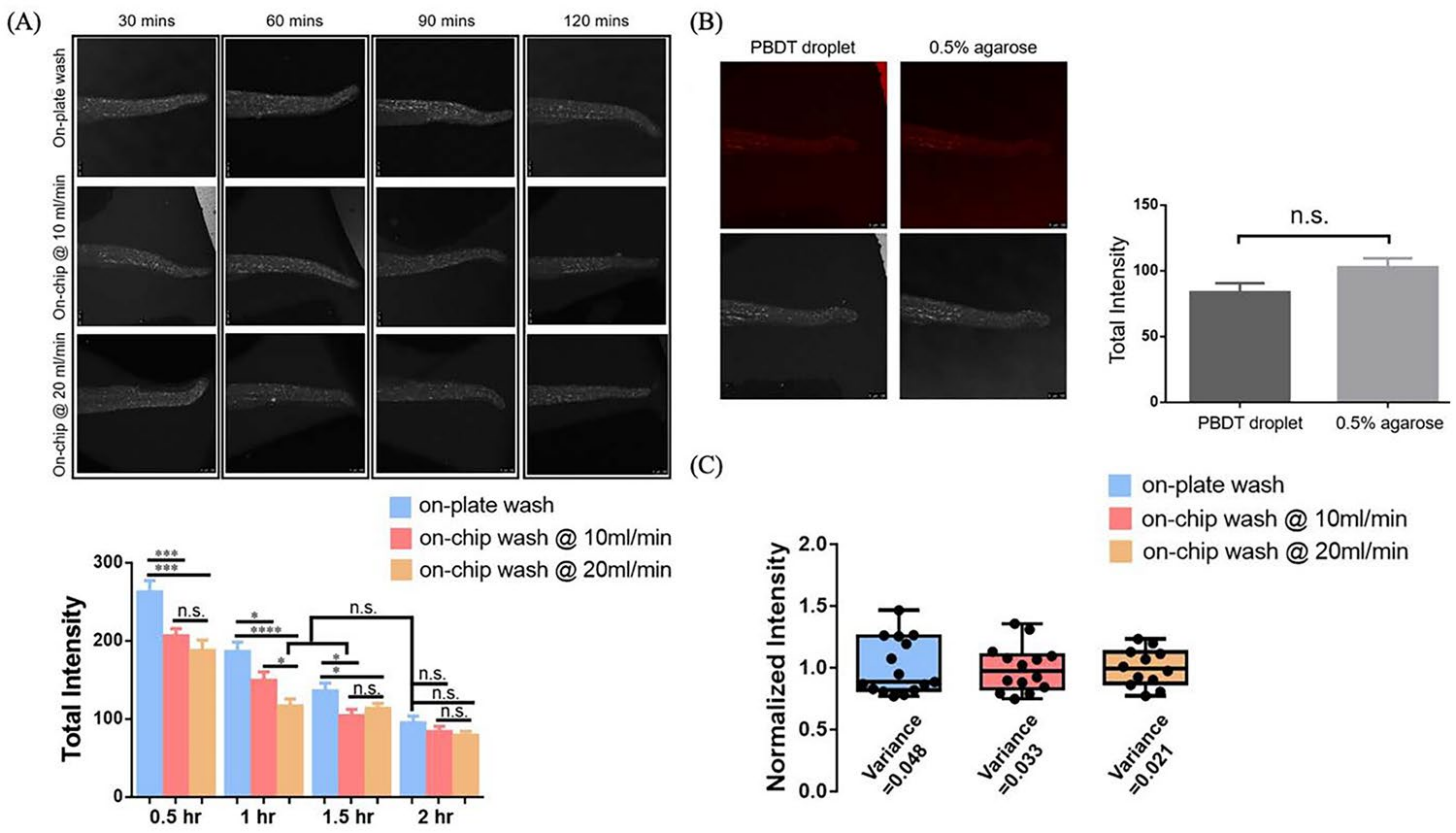
On-chip versus. on-plate whole mount zebrafish Caspase-3 ABS. **A** Top: gray scale images of the tail and trunk for 34 hpf UV treated zebrafish embryo after Caspase-3 ABS. (Vertical axis: on-plate ABS and on-chip ABS at different washing flowrates; Horizontal axis: different PBDT washing times). Bottom: The quantified signal levels for on-plate ABS (measured in 0.5% agarose) and on-chip ABS (measure on-chip in PBDT droplets) at different PBDT washing times and flowrates. (N $\ge$ 3, n $\ge$ 10, error bar: + SEM. **B** The signal levels for the same set of embryos measured on-chip and in 0.5% agarose, error bar: + SEM. **C** The experiment variances for on-chip and on-plate whole mount zebrafish Caspase-3 ABS (N $\ge$ 12)

Based on the experiment results, the washing process was found to be accelerated using the device as significant intensity differences were found at 30 mins, 60 mins, and 90 mins between the on-plate and the on-chip washings, Fig. 8A, bottom panel. Also, the samples were found already sufficiently washed at 60 mins when the on-chip washing flowrate is 20 ml/min. In addition, a significant intensity difference was found between the two tested on-chip washing flowrates at the 60 mins which implied a higher on-chip washing flowrate can speed up the washing process. The two tested on-chip washing flowrates showed insufficient washing at 30 mins as the intensities for both flowrates were significantly higher than the control and no significant intensity difference can be distinct between them. Regarding the image taking environments, the intensity measured from the PBDT droplets inside the chip has a slightly decreased signal compared to the intensity measured in the 0.5% agarose gel. This is likely due to the power attenuation of the fluorescent laser when penetrating through the PDMS layer on top of the device. Despite this, the signal difference between the on-chip and in agarose measurements is not significant, Fig. 8B. We next normalized the intensity of all the experimental results measured by respective means and the consistency of the device-based procedure is found higher than that of the plate-based procedure, Fig. 8C. Also, the consistency of the assay seemed to be improved when applying higher on-chip washing flowrate.

All in all, the milli fluidic-based whole mount zebrafish ABS outperforms the conventional plate-based manual approach by reducing both manual steps and time while increasing the consistency of the results. This highlights the benefits of miniaturization and mechanization of zebrafish assays.

## Discussion and conclusions

Zebrafish-on-a-chip may have experienced significant growth lately, but it is still in the early development phase (Li et al. 2014; Yang et al. 2016). The needs for optimizing and automating the time consuming and labor-intensive procedures such as whole mount zebrafish ABS and ISH is still largely unfulfilled. To fill the gap, we developed a multi-depth spiral milli fluidic device that can trap, immobilize, and perform the whole mount Caspase-3 ABS on the chorion-less zebrafish embryo. The device was fabricated by using a 3D printer assisted rapid prototyping method. Remarkably, the commercial leveled 3D printer and photo resin were used in making the master mold in this study. Compared with the fabrication methods reported previously (Fuad et al. 2017; Yang et al. 2016), our method is more economical in prototyping zebrafish-on-a-chip devices and can be easily adapted by small budget labs.

The multi-depth spiral device developed in the study uses the classic hydrodynamic trapping mechanism yet a chaotic trapping process to trap chorion-less zebrafish embryos in the close-loop pumping system. The device showed a trap usage rate that is comparable to the previously reported zebrafish embryo trapping platforms (Akagi et al. 2012; Fuad et al. 2017), but with a more convenient operating procedure as multiple embryos can be loaded into the chip at the beginning. However, in this study only one embryo size (i.e., UV treated chorion-less 36 hpf zebrafish embryos with a body length around 2 mm) were tested in the trapping validation experiment. Although the device has proven to be effective in trapping this type of embryos, using embryos with different body size or shape (i.e., embryo with intact chorion) may result in different trapping performance and the channel dimension also need to be modified accordingly to trap embryo with intact chorion. Follow-up experiments are needed in the future to test the limitations of our device in trapping embryos with different sizes and shapes. In addition, the orientation preference (i.e., head point inward) found when trapping the cone-shaped fixed zebrafish embryo has simplified the embryo loading process as no additional embryo orientation adjustment is needed prior the trapping. However, we can only provide a superficial explanation for this phenomenon in the current study. To comprehensively understand this phenomenon, further investigation using more powerful simulation tools is needed in the future. Besides, we hope this phenomenon can provide some insights for the trapping and sorting of non-spherical particles in other fluidic devices. Furthermore, the trapped embryos were found to be encapsulated in the droplets after draining out the buffer. The encapsulation of embryos in droplets makes the device become portable and allows the device to access various imaging platforms after trapping. This feature, which has not been reported in similar devices, may have potential to be used for the applications such as embryonic toxicity tests, drug screening, metabolite analysis, hypoxia study etc.

In this study, the complete procedure of the whole mount zebrafish ABS was performed and optimized on a zebrafish-on-a-chip device for the first time. We proved that the washing process in the whole mount zebrafish Caspsae-3 ABS procedure can be accelerated by a higher perfusion flowrate. We believe this finding can also be applied to other types of whole mount zebrafish ABS procedures or even more complicated ISH procedures.

The current device and system setting still have limitations that need to be optimized in the future. First, the - milli fluidic system requires more reagent volume (i.e., ~ 2 times for washing and ~ 6 times for staining) compared to the on-plate procedure due to off-chip volumes contributed by the tubing and pump. The large reagents requirement could be reduced by shortening or reducing the size of the tubing. Also, the effect of large reagent consumption can be minimized by conducting large scale tests (i.e., connect multiple devices in series) or by reusing the reagents (Fuqua et al. 2021). Second, although the multi-depth device can prevent large- or micro- sized bubbles from entering the trap, the medium sized bubbles may still occasionally enter the traps during the perfusion. This is the major cause of bias in the study as the trapped bubbles will not only affect the flow through the traps but also block the embryo during the imaging. Therefore, a special bubble trapping or breaking device (Fu et al. 2011) could be added at the device inlet to trap the medium size bubbles or break the medium size bubble into micro bubbles. Finally, the current procedure still requires the operator to manually load the embryos and switch the buffers during each step. The manual buffer switching process can be eliminated by integrating the device with automated imaging and liquid handling platforms (Fuqua et al. 2021) in the future to reach the degree of “sample-in-and-answer-out”.

### Supplementary Information

Below is the link to the electronic supplementary material.

Supplementary file1 (MP4 382843 KB)

Supplementary file2 (MP4 231920 KB)

Supplementary file3 (MP4 51576 KB)

Supplementary file4 (MP4 90106 KB)

Supplementary file5 (MP4 55063 KB)

Supplementary file6 (MP4 38682 KB)

Supplementary file7 (DOCX 3611 KB)
