# Supplementary material for: A multi-depth spiral milli fluidic device for whole mount zebrafish antibody staining
Source: Biomed Microdevices. 2023 Aug 15;25(3):30. doi: 10.1007/s10544-023-00670-2 (PMC10427545; doi:10.1007/s10544-023-00670-2)
Supplement: Supplementary file 7 — Supplementary file7 (DOCX 3611 KB) [file 10544_2023_670_MOESM7_ESM.docx]

**Supplementary Information**

**A Multi-depth Spiral Milli Fluidic Device for Whole Mount Zebrafish Antibody Staining**

Songtao Ye^1^, Wei-Chun Chin^1,2, *^, and Chih-Wen Ni^1,2^

^1^ Department of Quantitative and Systems Biology, University of California Merced.

^2^ Department of Bioengineering, University of California Merced.

*Corresponding author: Wei-Chun Chin, Email: [wchin2@ucmerced.edu](mailto:wchin2@ucmerced.edu), ORCID: 0000-0003-4881-9085

**
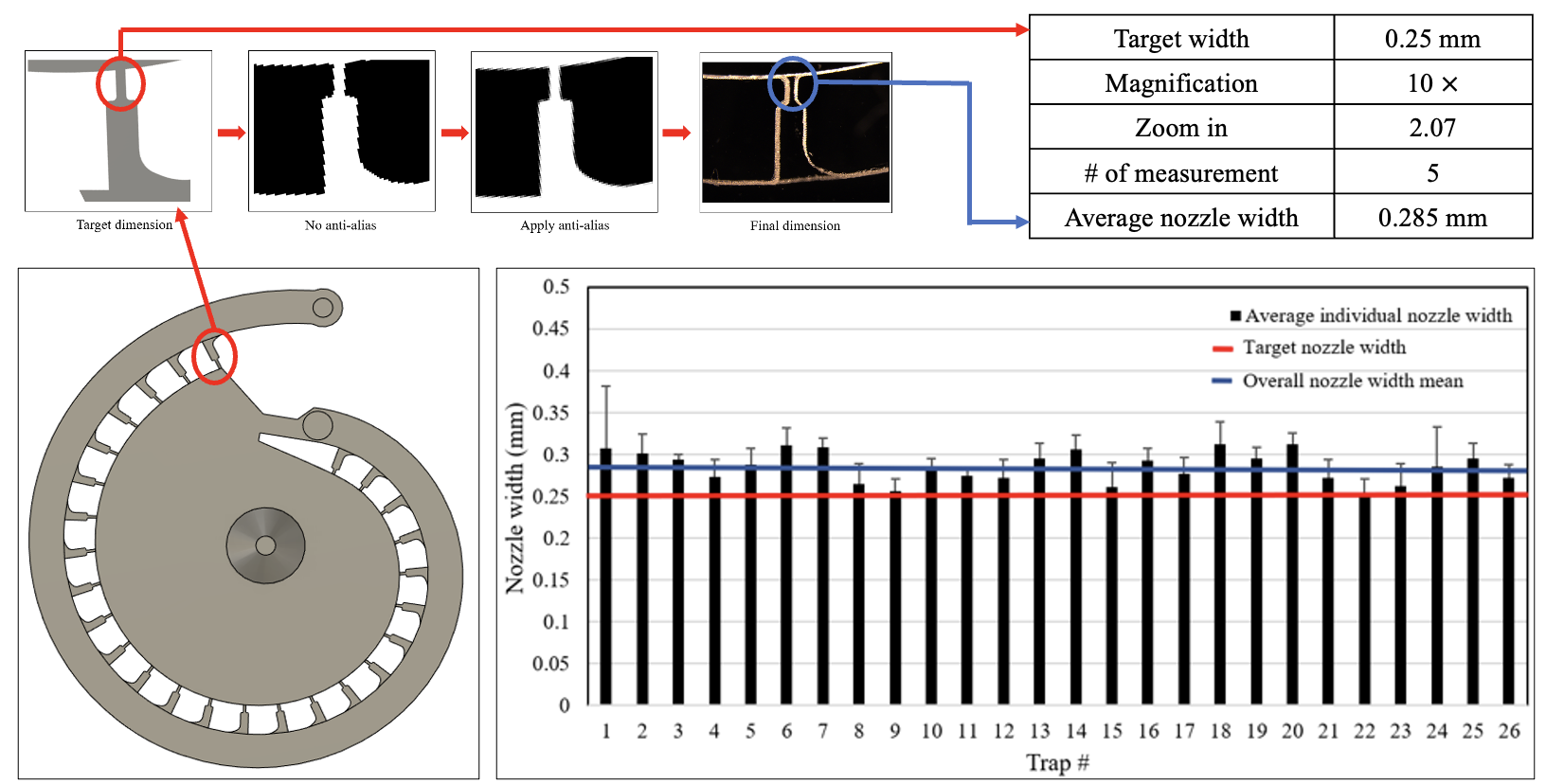
**

**Fig. 1S.** The device fabrication inspection. **Top left**: The anti-aliasing algorithm used to reduce the jaggedness in the channel wall. **Top right**: The table for the target nozzle width, measured nozzle width, and the settings for the inspection. **Bottom left**: The CAD drawing of the multi-depth spiral device. **Bottom right**: The result for the nozzle width inspection (N = 5).


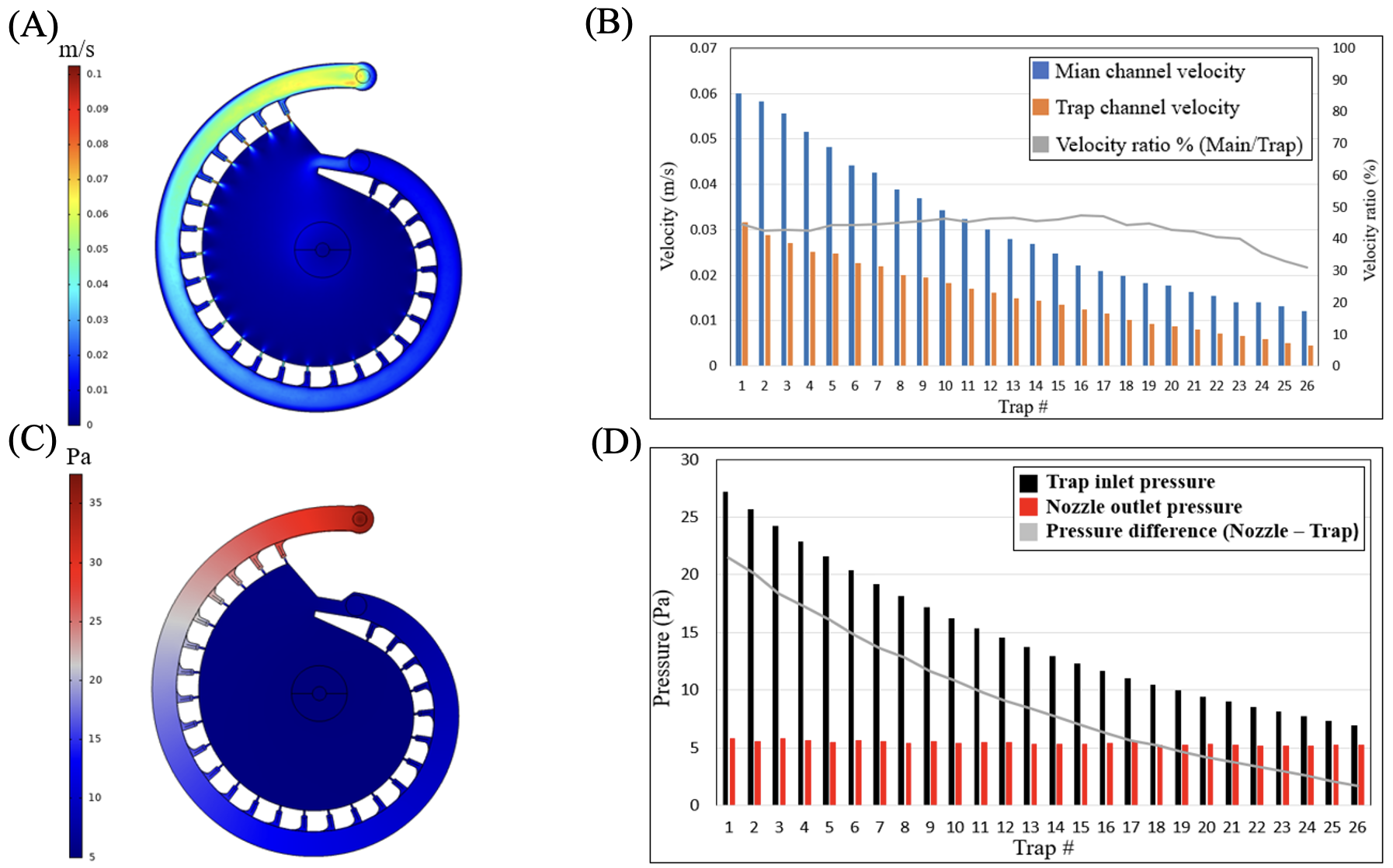


**Fig. 2S.** The CFD analysis for the device design. **A)** The velocity field for the multi-depth spiral device at the beginning of the trapping when the inlet flowrate is 10 ml/min. **B)** The maximum velocity profile measured in each individual trap and at the near main channel sections. **C)** The pressure field for the multi-depth spiral device at the beginning of the trapping when the inlet flowrate is 10 ml/min. **D)** The pressure profile measured at each trap inlets and at the corresponding nozzle outlets.


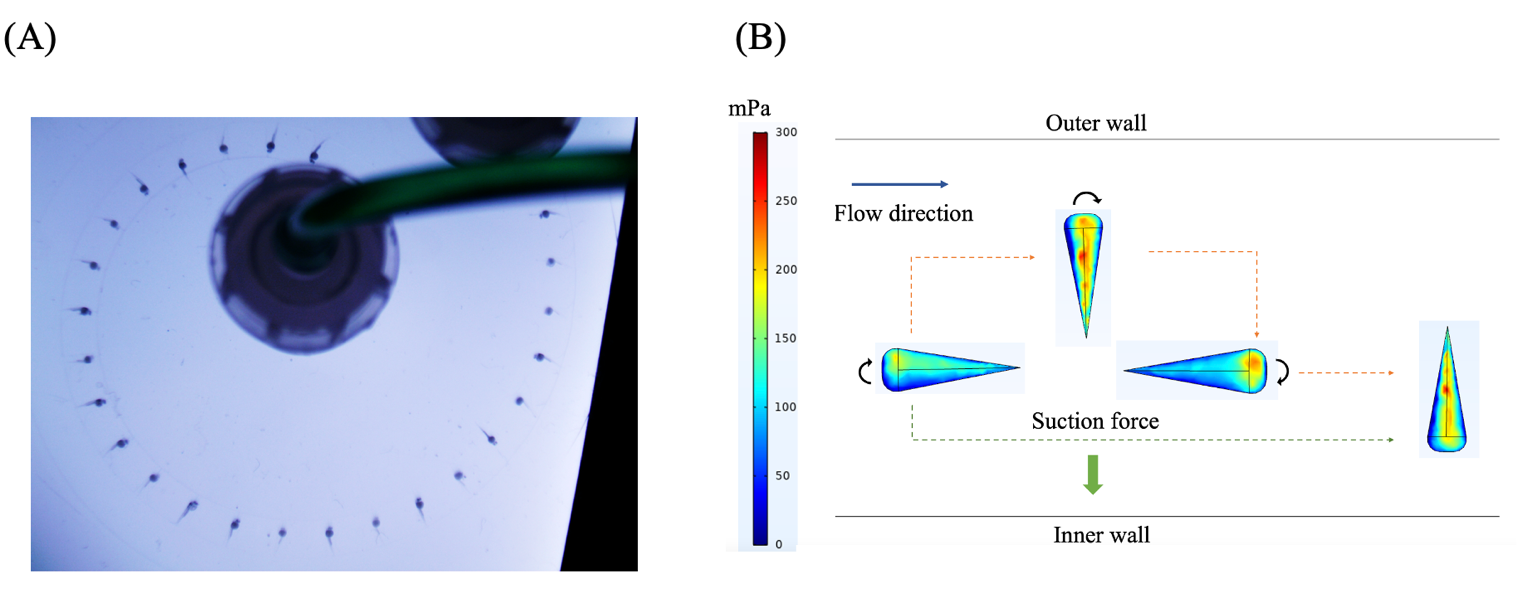


**Fig. 3S.** Zebrafish embryo body orientation patterns after trapping. A) Image showing most of trapped zebrafish embryos have their heads pointed inward. B) The shear stress distribution on the 4 potential body orientations of the zebrafish embryo in the main channel (top view). The blue arrow indicates the flow direction. The black arrow indicates the direction of potential embryo body rotation when there is no suction force. The green arrow indicates the direction of hydrodynamic suction force.


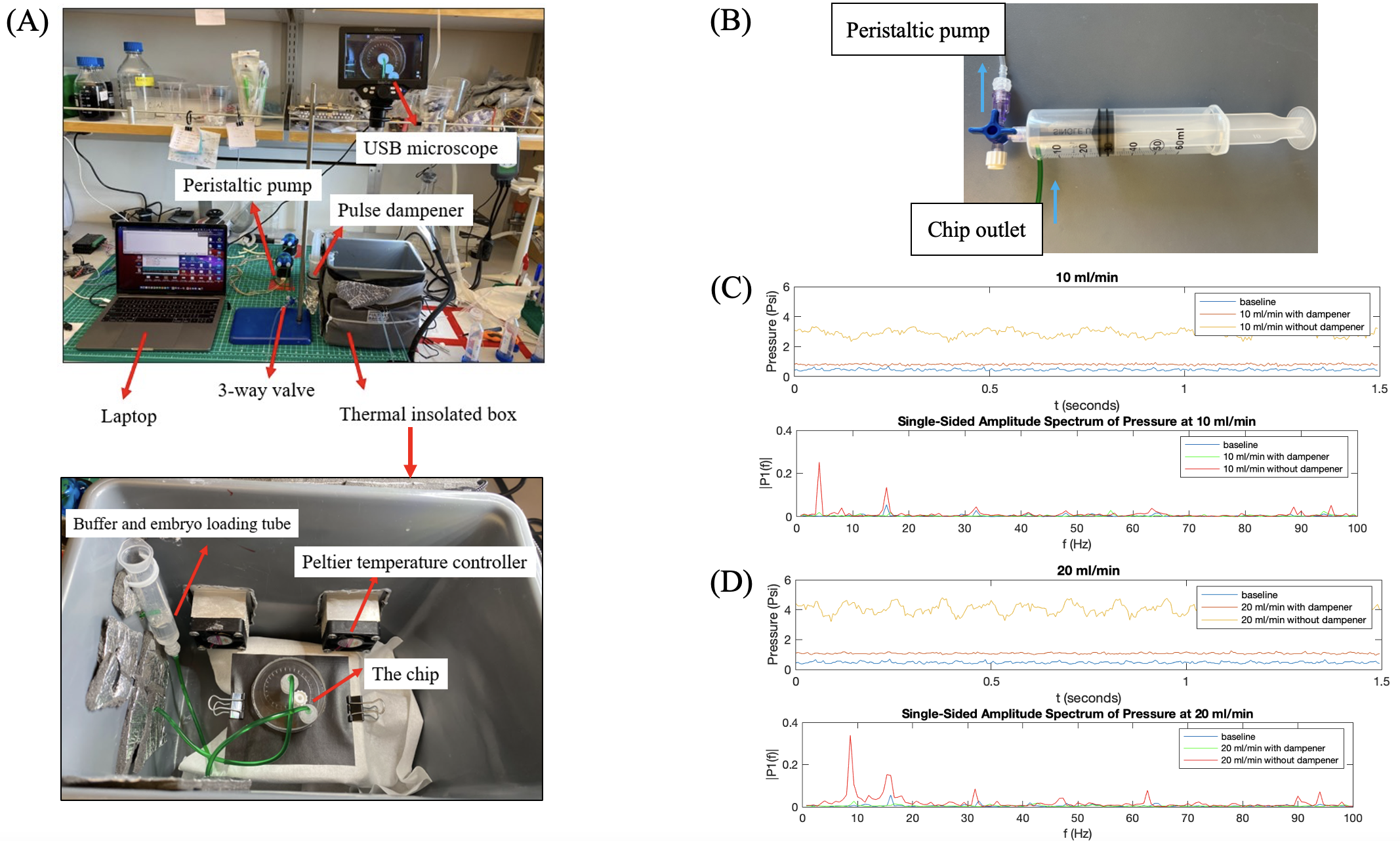


**Fig. 4S.** The overall system setups and the homemade pulse dampener. **A)** **Top:** The overall setups for the zebrafish embryo trapping and staining system. **Bottom:** The components inside the thermal insulated box. **B)** The picture of the homemade pulse dampener using a 60 ml lure lock syringe and a 2-way valve. The air volume inside the syringe is set to be 26 ml. **C) Top:** The pressure profile measured at the inlet of the peristaltic pump with and without the pulse dampener at 10 ml/min flowrate. **Bottom:** the pressure profile after fast Fourier transform. **D) Top:** The pressure profile measured at the inlet of the peristaltic pump with and without the pulse dampener at 20 ml/min flowrate. **Bottom:** the pressure profile after fast Fourier transforms.
